# Pregnant Women’s Intentions and Subsequent Behaviors Regarding Maternal and Neonatal Service Utilization: Results from a Cohort Study in Nyanza Province, Kenya

**DOI:** 10.1371/journal.pone.0162017

**Published:** 2016-09-13

**Authors:** Andreea A. Creanga, George Awino Odhiambo, Benjamin Odera, Frank O. Odhiambo, Meghna Desai, Mary Goodwin, Kayla Laserson, Howard Goldberg

**Affiliations:** 1 Department of International Health, Johns Hopkins Bloomberg School of Public Health, Baltimore, MD, United States of America; 2 International Center for Maternal and Newborn Health, Johns Hopkins Bloomberg School of Public Health, Baltimore, MD, United States of America; 3 Centre for Global Health Research, Kenya Medical Research Institute, Kisumu, Kenya; 4 Division of Parasitic Diseases and Malaria, Center for Global Health, Centers for Disease Control and Prevention, Atlanta, GA, United States of America; 5 Division of Reproductive Health, National Center for Chronic Disease Prevention and Health Promotion, Centers for Disease Control and Prevention, Atlanta, GA, United States of America; 6 Division of Global Health Protection, Center for Global Health, Centers for Disease Control and Prevention, Atlanta, GA, United States of America; National Institute of Health, ITALY

## Abstract

Higher use of maternal and neonatal health (MNH) services may reduce maternal and neonatal mortality in Kenya. This study aims to: 1) prospectively explore women’s intentions to use MNH services (antenatal care, delivery in a facility, postnatal care, neonatal care) at <20 and 30–35 weeks’ gestation and their actual use of these services; 2) identify predictors of intention-behavior discordance among women with positive service use intentions; 3) examine associations between place of delivery, women’s reasons for choosing it, and birthing experiences. We used data from a 2012–2013 population-based cohort of pregnant women in the Demographic Surveillance Site in Nyanza province, Kenya. Of 1,056 women completing the study (89.1% response rate), 948 had live-births and 22 stillbirths, and they represent our analytic sample. Logistic regression analysis identified predictors of intention-behavior discordance regarding delivery in a facility and use of postnatal and neonatal care. At <20 and 30–35 weeks’ gestation, most women intended to seek MNH services (≥93.9% and ≥87.5%, respectively, for all services assessed). Actual service use was high for antenatal (98.1%) and neonatal (88.5%) care, but lower for delivery in a facility (76.9%) and postnatal care (51.8%). Woman’s age >35 and high-school education were significant predictors of intention-behavior discordance regarding delivery in a facility; several delivery-related factors were significantly associated with intention-behavior discordance regarding use of postnatal and neonatal care. Delivery facilities were chosen based on proximity to women’s residence, affordability, and service quality; among women who delivered outside a health facility, 16.3% could not afford going to a facility. Good/very good birth experiences were reported by 93.6% of women who delivered in a facility and 32.6% of women who did not. We found higher MNH service utilization than previously documented in Nyanza province. Further increasing the number of facility deliveries and use of postnatal care may improve MNH in Kenya.

## Introduction

Kenya’s maternal and neonatal mortality rates remain at unacceptably high levels. Each year, approximately 6,300 women die from pregnancy complications, resulting in a maternal mortality ratio of 400 deaths per 100,000 live births [1]; also, 27 of every 1,000 infants born alive die during their first month [2]. When examining the country’s success in achieving Millennium Development Goals 4 and 5 by 2015, we find that progress has been made to reduce overall infant and under-five child mortality [3], but not neonatal [3] or maternal mortality [1]. Clearly, the latter two indicators are interconnected.

Historical studies show that maternal mortality decreased considerably in developed countries at the beginning of the 19th century, due to a combination of factors, among which were the introduction of penicillin, the large scale availability of blood transfusions, and improved obstetric care [4]. The World Health Organization recommends that all women have access to a skilled birth attendant for antenatal care, delivery, and postpartum care [5], and it has been estimated that the presence of a skilled attendant at birth can prevent up to one-third of maternal deaths [6]. Skilled birth attendants are key to protecting the health of newborns, since most perinatal deaths occur during labor and delivery or within the first 48 hours thereafter [2].

Decisions to use maternal and neonatal health (MNH) services in facilities with skilled medical personnel are a function of both individual and health system factors. The 3-delay framework proposed by Thaddeus and Maine [7] recognizes three levels of barriers to obstetric care: delays in decisions to seek care; delays in arrival at health facilities; and delays in the provision of adequate care. Thus, strategies to promote the utilization of obstetric services should extend beyond the health system to include factors influencing women’s decision-making and health seeking behaviors, as well as the socio-economic, cultural, political, and religious contexts in the communities where they live. Of all these factors, those related to decision-making regarding the use of health services are the most difficult to assess. The Theory of Planned Behavior [8] addresses the impact of cognitive components on both behavioral intentions and actual behaviors. According to this theory, individuals' attitudes toward a certain behavior (i.e. use of health services), [the norms representing] their perception of other people's view of such behavior, and their perceived ability to perform the behavior strongly influence their behavioral intentions; these factors may further lead to performance or nonperformance of the behavior. Prospective studies have shown that intentions typically account for 20%-40% of the variance in social and health behaviors [9–13]. Although this percentage is not small, it indicates that a number of people do not enact their positive intentions to perform a particular behavior. Given the limited use of MNH services in Kenya (e.g. 42.6% facility delivery, 47.0% postnatal care, 55.9% treatment of respiratory infections among children under-five) [14], it is important to examine both intentions and behaviors vis-à-vis MNH care utilization to identify ways to increase access to and use of these services.

The objectives of this study are to: 1) prospectively assess women’s intentions to use MNH services at two different times during pregnancy and compare this to their actual use of these services during the index (i.e. current) pregnancy; 2) identify predictors of intention-behavior discordance regarding use of MNH services among women with positive service use intentions; and 3) examine associations between place of delivery and women’s reasons for choosing it, reasons for not delivering in their “preferred” place of delivery, and overall birthing experiences.

## Materials & Methods

We used data from the Maternal and Neonatal Health Care Utilization Study conducted in two regions (Asembo and Gem) covered by the Health and Demographic Surveillance System (HDSS) in Kisumu, Nyanza province, Kenya. The study employed a large population-based cohort of pregnant women in the two regions to identify ways to increase the use of MNH services in Kenya. The study population is comprised of all pregnant women residing in HDSS villages within a 5 km radius of Lwak Hospital in Asembo and a 5 km radius of Dienya Health Centre in Gem who were identified by trained HDSS community interviewers at ≤20 weeks’ gestation between January 2012 and December 2013. Women who did not know the exact first day of their last menstrual period or their gestational age were referred for free clinical determination of gestational age by ultrasound at Lwak Hospital (if residing in Asembo study villages) or Dienya Health Centre (if residing in Gem study villages). Consenting women were interviewed through household surveys three times: upon enrollment at ≤20 weeks’ gestation (baseline interview); between 30 and 35 weeks’ gestation (follow-up interview), and within 6 weeks of the end of pregnancy regardless of the outcome (endline interview). Written informed consent for participation in the study was obtained from all study subjects. The study protocol was reviewed and approved by Institutional Review Boards at the U.S. Centers for Disease Control and Prevention in Atlanta, GA and at the Kenya Medical Research Institute in Nairobi, Kenya.

Women were interviewed by trained interviewers who did not reside in the same villages as the women they were interviewing. The baseline questionnaire collected key socio-demographic information and included each respondent’s pregnancy history, self-reported pregnancy complications, overall health status, and intended use of antenatal, delivery, and postnatal care services for themselves as well as neonatal care for their infants. During follow-up interviews, women were asked about pregnancy complications developed since the baseline interview and their intention to use/continue to use antenatal care, as well as delivery and postnatal care services for themselves and neonatal care for their infants. At the endline interview, information was obtained on pregnancy outcomes, the actual use of MNH services, women’s reasons for choosing their respective place of delivery and, when appropriate, reasons for not choosing their “preferred” place of delivery, information about their intention to deliver in the same place if ever pregnant again, and their birth experience ratings. Information gathered is in line with Anderson’s behavioral model outlining predisposing, enabling, perceived need, and service-related factors that can contribute to decision-making around care seeking and actual care seeking [15].

For our analysis, we first compared women’s socio-demographic and health-related characteristics by study site (Asembo vs Gem) using chi-square tests for proportions and t-tests for means. The socio-demographic characteristics of interest were: age (<20, 20–24, 25–29, 30–34, 35+ years); parity (primipara vs multipara); marital status (married/in union, monogamous; married/in union, polygamous; single); education level (<5, 5–8, 9+ completed years); and religion (protestant, Catholic, other). Health-related characteristics of interest for this analysis were: gestational ages at baseline and follow-up interviews as well as at the end of the pregnancy; self-rated overall health status (very poor/poor, neither poor nor good, good/very good); presence of a (known) chronic medical condition, including HIV/AIDS (yes/no); main household decision-maker(s) regarding health issues (woman herself, her husband/partner, both, other household member); pregnancy complications assessed prospectively at baseline, if developed between baseline and follow-up interviews, at follow-up, if developed between follow-up and endline interviews, and at any time during the index pregnancy; whether or not the woman sought care for index pregnancy complications by follow-up or endline interview times; presence of delivery complications at index delivery (yes/no); mode of delivery (vaginal vs cesarean); index pregnancy outcome (live birth vs stillbirth); and self-reported index birth experience (good/very good, poor/very poor/neither good nor poor). A variable of key interest for this analysis was women’s knowledge about whether free maternal health services were available to them. All women enrolled in Asembo villages were offered free antenatal care services at Lwak Hospital through the study, while women enrolled in Gem villages were not. On June 1, 2013, the Government of Kenya implemented a program that offers free maternity care (both antenatal and delivery care) in public sector facilities to all Kenyan women. Thus, we used the interview place (Asembo vs Gem) and date (before or after June 1, 2013) to create variables for women’s knowledge of available free antenatal care at the time of baseline and follow-up interviews and knowledge of available free delivery care at the time of follow-up and endline interviews.

For the first study objective, we examined the following outcomes: women’s intentions at baseline to use antenatal, delivery, and postnatal care; women’s intentions at follow-up to use/continue to use antenatal, delivery, and postnatal care; women’s actual use of antenatal, delivery, and postnatal care during the index pregnancy; women’s intentions to deliver in the same place if ever pregnant again; and, among women with a live birth, their intentions at baseline and follow-up to use neonatal health services for their infants and their actual use of these services for their infants. All outcome variables were dichotomous (yes/no).

There was little variability in women’s intentions and behaviors regarding use of antenatal care. Thus, for the second study objective, we restricted the sample to women with positive intentions to use delivery, postnatal, and neonatal care and aimed to identify predictors of discordance between women’s (positive) intentions and their actual use of these three types of services. For each of the three types of service, we created two distinct dichotomous outcome variables: (O1) discordance between positive intention at baseline to use services and not using the services per endline reports; and (O2) discordance between positive intention at follow-up to use services and not using the services per endline reports. We fitted logistic regression models for all six outcomes, adjusting for the following covariates: the five socio-demographic characteristics noted above; self-rated health status; presence of at least one chronic medical condition of the following: preexisting diabetes, chronic hypertensive disease, chronic heart disease, chronic respiratory disease, chronic renal disease, chronic liver disease, HIV/AIDS; main household decision-maker(s) on health issues; and study site. Models fitted for O1 were also adjusted for gestational age at baseline and at the end of pregnancy, reports of pregnancy complications at present at baseline or developed between baseline and endline interviews, and reports of women seeking care for pregnancy complications by endline; models fitted for O2 were also adjusted for gestational age at follow-up and at the end of pregnancy, reports of pregnancy complications at follow-up or developed between follow-up and endline interviews, and reports of women seeking care for pregnancy complications by endline. Knowledge of free delivery services at follow-up was added in facility delivery models fitted for O1, while knowledge of free delivery services at endline was added in facility delivery models fitted for O2. In addition, postnatal and neonatal care models fitted for both O1 and O2 were adjusted for presence of delivery complications, mode of delivery, index pregnancy outcomes, and birth experience ratings.

Lastly, chi-square tests were used to compare: 1) women who delivered in and out of health facilities, and 2) women who would, were unsure, and would not deliver in the same place if pregnant again with regard to reasons for choosing their specific place of delivery, reasons for not delivering in their “preferred” place of delivery, and their self-rated birth experiences.

## Results

Overall, 593 women were enrolled in Asembo villages and 592 women in Gem villages. Of these, 96 women were lost to follow-up and 3 women died from pregnancy complications before follow-up interviews; an additional 29 women were lost to follow-up and 1 woman died between follow-up and endline interviews. Thus, the overall study response rate was 89.1%, lower in Gem (85.1%) than in Asembo (93.8%) villages. Among the 1,056 women who completed the study, 948 had a live birth, 22 a stillbirth, and 86 a spontaneous or an induced abortion. Given the objectives of this analysis, the sample was restricted to the 970 women who had a birth (live birth or stillbirth).

The socio-demographic characteristics of the 970 respondents did not differ significantly by study site (Asembo vs Gem) (Table 1). Overall, over 70% were younger than 30 years; almost 85% were multiparas; about 4 of every 5 were married; about one-fifth completed secondary education. For Health-related characteristics, the mean gestational age at baseline was slightly lower for women enrolled in Asembo (12.3 weeks) than in Gem (14.0 weeks), but this difference did not carry over to follow-up interviews. Almost one-quarter (24.0%) of women in Asembo, compared with 9.8% in Gem, reported having at least one of the chronic medical conditions considered, with chronic respiratory disease and chronic hypertensive disease being the most commonly reported. In line with these reports, a lower proportion of women in Asembo (64.4%) than in Gem (71.6%) rated their overall health as good or very good. Also, at baseline, a higher percentage women in Asembo (25.3%) than in Gem (15.6%) self-reported having one or more pregnancy complications, while 7.3% of women in Asembo versus 14.5% in Gem reported complications during labor and delivery. Compared to 70.7% of women in Gem, 88.0% of women in Asembo rated their index birth experience as good or very good.

**Table 1:** Study population characteristics.

Characteristics	Asembo N = 509	Gem N = 461	p-value*	Total N = 970
***Socio-demographic characteristics***				
Age-group (years; %)			0.253	
<20	18.9	23.2		20.9
20–24	28.1	28.9		28.5
25–29	24.2	23.4		23.8
30–34	18.9	14.1		16.5
35+	10.2	10.4		10.3
Parity (%)			0.625	
Primipara	15.1	16.3		15.7
Multipara	84.9	83.7		84.3
Marital status (%)			0.33	
Married/in union, monogamous	65	68.6		66.7
Married/in union, polygamous	12	12.4		12.2
Single	23	19.1		21.1
Education (years; %)			0.252	
<5	3.7	5.9		4.7
5–9	73.9	73.8		73.8
>9	22.4	20.4		21.4
Religion (%)			0.232	
Protestant	65.6	66.2		65.9
Catholic	17.1	13.7		15.5
Other	17.3	20.2		18.7
***Health-related characteristics***				
Mean (std dev) gestational age (wks)				
Baseline	12.3 (4.5)	14.0 (4.8)	<0.001	13.1 (4.8)
Follow-up	30.3 (0.7)	30.4 (0.9)	0.051	30.4 (0.8)
End of pregnancy	37.2 (3.7)	37.5 (3.5)	0.121	37.3 (3.6)
Self-rated health status (%)			0.004	
Very poor/Poor	10.4	5		7.8
Neither poor nor good	25.2	23.4		24.3
Good/Very good	64.4	71.6		67.8
Has (known) chronic medical condition(s) (%)**	24	9.8	<0.001	17.2
Main health decision-maker (%)			<0.001	
Herself	43.2	55.5		49.1
Husband	30.8	18.2		24.9
Both	6.7	19.5		12.8
Other	19.3	6.7		13.3
Self-reported pregnancy complications (%)				
Baseline reports	25.3	15.6	<0.001	20.7
Follow-up reports	37.5	31.7	0.056	34.7
Developed between baseline & follow-up	12.2	16.1	0.083	14
Developed between baseline & endline	20.4	25.4	0.067	22.8
Developed between follow-up & endline	8.3	9.3	0.554	8.8
Any during pregnancy	45.8	41	0.134	43.5
Sought care for pregnancy complications (%)				
By follow-up time	11	11.1	0.976	11
By endline	23.2	21.9	0.636	22.6
Delivery complications (%)	7.3	14.5	<0.001	10.7
Vaginal delivery (%)	97.1	97.8	0.126	97.4
Pregnancy outcome (%)			0.289	
Live birth	97.3	98.3		97.7
Stillbirth	2.7	1.7		2.3
Self-reported birth experience (%)			<0.001	
Good/very good	88	70.7		79.5
Poor/very poor/neither good nor poor	12	29.1		20.5
Knowledge of free services (%)***				
Antenatal care				
At baseline	100	0	<0.001	47.5
At follow-up	100	14.3	<0.001	59.3
Delivery care				
At follow-up	11.8	14.3	0.242	13
At endline	33.4	32.3	0.721	32.9

Notes:

*Based on chi-square tests or t-tests;

**At least one of the following conditions: preexisting diabetes, chronic hypertensive disease, chronic heart disease, chronic respiratory disease, chronic renal disease, chronic liver disease, HIV/AIDS

***Study participants in Asembo were offered free antenatal care through the study. In addition, on June 1 2013, the Government of Kenya made maternity services free in all public sector facilities, thus impacting study participants interviewed after this date in both study sites.

**Fig 1:**
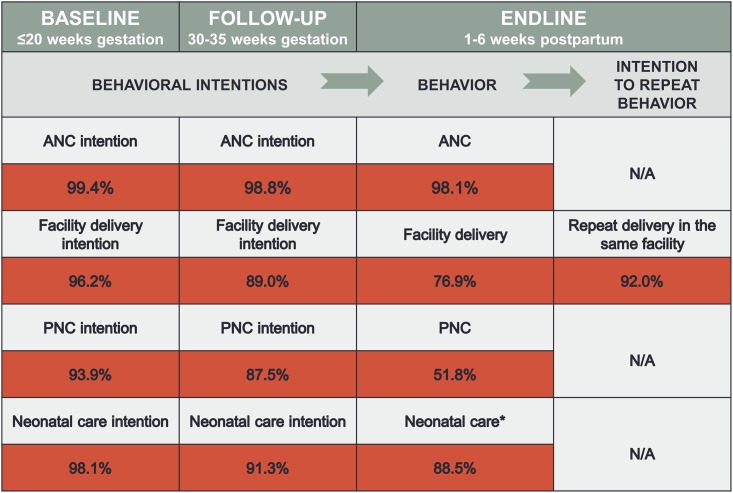
Intentions, behaviors and intentions to repeat behaviors regarding use of maternal and neonatal health services. *Notes*: ANC, antenatal care; PNC, postnatal care; *of those with a live birth.

At both baseline and follow-up, the overwhelming majority of women intended to use antenatal care (99.4% and 98.8%, respectively), deliver in a health facility (96.2% and 89.0%, respectively), seek postnatal (93.9% and 87.5%, respectively) and neonatal (98.1% and 91.3%, respectively) care (Fig 1). Actual service use was high for antenatal (98.1%) and neonatal (88.5%) care, but lower for delivery in a facility (76.9%) and postnatal care (51.8%).

We found three significant predictors of discordance between baseline or follow-up reports of intending to deliver in a health facility and women’s actual delivery place (Table 2): women’s age (≥35 vs. 25–29 years), education (≥9 vs. 5–8 years), and living in households where someone other than the woman or her husband/partner made health-related decisions. Among women who intended to deliver in a health facility at the time of follow-up interviews, knowledge of the availability of free delivery care significantly increased the odds of their doing so. Notably, women in Gem had 1.7–1.8 times higher odds than those in Asembo to not follow through on their baseline or follow-up intentions to deliver in a health facility.

**Table 2:** Predictors of discordance between women’s intentions and behaviors regarding facility delivery: Kenya, 2013.

Characteristics	Facility delivery (endline) vs facility delivery intention (baseline) N = 933	Facility delivery (endline) vs facility delivery intention (follow-up) N = 863
	OR (95% CI)	
Age-group (25–29 = ref)		
<20	0.99 (0.53, 1.86)	0.78 (0.38, 1.60)
20–24	1.05 (0.66, 1.66)	1.09 (0.68, 1.76)
30–34	1.15 (0.67, 1.96)	1.23 (0.71, 2.13)
35+	2.32 (1.32, 4.08)**	2.27 (1.26, 4.12)**
Primipara (multipara = ref)	0.70 (0.35, 1.41)	0.70 (0.31, 1.61)
Marital status (married/in union, monogamous = ref)		
Married/in union, polygamous	1.06 (0.65, 1.73)	1.02 (0.61, 1.71)
Single	1.35 (0.79, 2.30)	1.37 (0.76, 2.48)
Education (5–8 years = ref)		
<5	0.82 (0.40, 1.72)	0.70 (0.31, 1.56)
>9	0.45 (0.28, 0.72)**	0.84 (0.30, 0.79)**
Religion (Protestant = ref)		
Catholic	0.98 (0.61, 1.56)	1.05 (0.63, 1.76)
Other	0.82 (0.53, 1.28)	0.71 (0.44, 1.15)
GA at baseline (weeks)	1..01 (0.98, 1.05)	
GA at follow-up (weeks)		0.97 (0.76, 1.23)
GA at end of pregnancy (weeks)	0.93 (0.89, 0.97)**	0.94 (0.88, 1.00)*
Self-rated health status (good/very good = ref)		
Neither poor nor good	0.81 (0.54, 1.21)	0.70 (0.46, 1.08)
Very poor/Poor	0.97 (0.50, 1.85)	1.07 (0.56, 2.06)
Has chronic medical condition (no = ref)	0.72 (0.45, 1.15)	0.79 (0.48, 1.29)
Main health decision-maker (herself = ref)		
Husband	1.03 (0.68, 1.57)	0.89 (0.57, 1.37)
Both	1.29 (0.80, 2.07)	1.02 (0.61, 1.73)
Other	0.45 (0.22, 0.94)**	0.34 (0.14, 0.81)**
Pregnancy complications at baseline (no = ref)	1.00 (0.62, 1.62)	
Pregnancy complications at follow-up (no = ref)		1.14 (0.73, 1.80)
Pregnancy complications developed between baseline & follow-up (no = ref)		
Pregnancy complications developed between baseline & endline (no = ref)	1.33 (0.80, 2.21)	
Pregnancy complications developed between follow-up & endline (no = ref)		1.53 (0.73, 3.23)
Sought care for pregnancy complications by follow-up time (no = ref)		
Sought care for pregnancy complications by endline (no = ref)	0.71 (0.42, 1.20)	0.71 (0.41, 1.22)
Knowledge of free delivery care services at follow-up (no = ref)		0.28 (0.12, 0.63)**
Knowledge of free delivery care services at endline (no = ref)	0.53 (0.36, 0.77)**	0.77 (0.50, 1.18)
Study site Gem (Asembo = ref)	1.80 (1.26, 2.58)**	1.74 (1.19, 2.53)**

Notes: OR, odds ratios; CI, confidence interval; all models adjusted for all the factors shown;

*p<0.10;

**p<0.05.

Regarding discordance between postnatal care use intention expressed at baseline and actual use of postnatal care services, two key predictors stand out—women with than without delivery complications had about half the odds of having intentions that differed from their actual use of services, while women whose birth experiences were poor, very poor, or neither poor nor good than good/very good had 1.5 times greater odds to contribute to this baseline intention—endline behavior discordance (Table 3). The higher the gestational age at follow-up interviews, the lower the intention-behavior discordance with regard to postnatal care, while the higher gestational ages at the end of pregnancy, the higher the discordance. Compared to married women in monogamous relationships, those in polygamous relationships were significantly less likely to exhibit such discordance; the same was true for women whose husbands/partners were the main health decision-makers. Women who had a stillbirth rather than a live birth were 4.4 times more likely to intend at their follow-up interview to obtain postnatal care and then not d

**Table 3:** Predictors of discordance between women’s intentions and behaviors regarding postnatal care: Kenya, 2013.

Characteristics	Postnatal care receipt (endline) vs postnatal care intention (baseline) N = 911	Postnatal care receipt (endline) vs postnatal care intention (follow-up) N = 849
	OR (95% CI)	
Age-group (25–29 = ref)		
<20	0.98 (0.58, 1.67)	0.70 (0.39, 1.26)
20–24	1.13 (0.78, 1.65)	1.20 (0.81, 1.76)
30–34	0.84 (0.54, 1.31)	0.92 (0.59, 1.46)
35+	1.17 (0.70, 1.94)	1.28 (0.76, 2.17)
Primipara (multipara = ref)	1.06 (0.60, 1.87)	1.16 (0.62, 2.18)
Marital status (married/in union, monogamous = ref)		
Married/in union, polygamous	0.65 (0.42, 1.01)*	0.61 (0.39, 0.96)**
Single	0.76 (0.48, 1.20)	0.82 (0.50, 1.36)
Education (5–8 years = ref)		
<5	0.87 (0.45, 1.66)	0.73 (0.37, 1.43)
>9	0.87 (0.63, 1.22)	0.84 (0.59, 1.19)
Religion (Protestant = ref)		
Catholic	1.30 (0.88, 1.92)	1.18 (0.78, 1.80)
Other	0.95 (0.67, 1.36)	0.81 (0.56, 1.17)
GA at baseline (weeks)	0.99 (0.96, 1.02)	
GA at follow-up (weeks)		0.77 (0.63, 0.93)**
GA at end of pregnancy (weeks)	1.07 (1.02, 1.11)	1.11 (1.04, 1.17)**
Self-rated health status (good/very good = ref)		
Neither poor nor good	1.10 (0.65, 1.85)	0.80 (0.57, 1.12)
Very poor/Poor	0.92 (0.59, 1.43)	0.90 (0.52, 1.55)
Has chronic medical condition (no = ref)	1.15 (0.79, 1.68)	1.16 (0.78, 1.71)
Main health decision-maker (herself = ref)		
Husband	0.74 (0.42, 1.04)*	0.64 (0.45, 0.92)**
Both	0.92 (0.59, 1.43)	0.82 (0.52, 1.30)
Other	1.10 (0.65, 1.85)	1.12 (0.64, 1.98)
Pregnancy complications at baseline (no = ref)	1.20 (0.81, 1.78)	
Pregnancy complications at follow-up (no = ref)		1.23 (0.85, 1.77)
Pregnancy complications developed between baseline & follow-up (no = ref)		
Pregnancy complications developed between baseline & endline (no = ref)	1.13 (0.73, 1.74)	
Pregnancy complications developed between follow-up & endline (no = ref)		1.31 (0.69, 2.47)
Sought care for pregnancy complications by follow-up time (no = ref)		
Sought care for pregnancy complications by endline (no = ref)	0.83 (0.54, 1.28)	0.69 (0.45, 1.08)*
Delivery complications (no = ref)	0.54 (0.33, 0.88)**	0.64 (0.38, 1.08)*
Cesarean (vaginal delivery = ref)	0.40 (0.14, 1.15)*	0.37 (0.12, 1.10)*
Stillbirth (live birth = ref)	2.09 (0.81, 5.40)	4.37 (1.32, 14.45)**
Poor/very poor/neither good nor poor self-reported birth experience (good/very good = ref)	1.50 (1.06, 2.12)**	1.40 (0.97, 2.02)*
Study site Gem (Asembo = ref)	0.90 (0.66, 1.22)	0.80 (0.59, 1.10)

Notes: OR, odds ratios; CI, confidence interval; all models adjusted for all the factors shown;

*p<0.10;

**p<0.05.

Regarding neonatal care, women’s higher gestational age at the end of pregnancy reduced the odds of discordance between baseline intention to seek and actual use of these services (Table 4). Conversely, a significant positive predictor of such intention-behavior discordance was women’s less than good, compared to good/very good, self-rated birth experience. We also identified two significant predictors of discordance between women’s intentions to use neonatal care during follow-up interviews and their corresponding behavior—women’s higher gestational age at the end of the pregnancy and having a cesarean versus a vaginal delivery.

**Table 4:** Predictors of discordance between women’s intentions and behaviors regarding neonatal care: Kenya, 2013.

Characteristics	Neonatal care receipt (endline) vs neonatal care intention (baseline) N = 952	Neonatal care receipt (endline) vs neonatal care intention (follow-up) N = 886
	OR (95% CI)	
Age-group (25–29 = ref)		
<20	0.91 (0.41, 2.04)	0.62 (0.23, 1.66)
20–24	0.77 (0.43, 1.38)	0.66 (0.35, 1.26)
30–34	0.87 (0.44, 1.69)	0.63 (0.29, 1.35)
35+	0.96 (0.45, 2.02)	1.06 (0.48, 2.34)
Primipara (multipara = ref)	0.61 (0.24, 1.52)	0.62 (0.21, 1.86)
Marital status (married/in union, monogamous = ref)		
Married/in union, polygamous	1.47 (0.80, 2.69)	1.28 (0.63, 2.59)
Single	1.66 (0.85, 3.22)	2.02 (0.95, 4.27)*
Education (5–8 years = ref)		
<5	1.08 (0.43, 2.69)	1.09 (0.40, 2.98)
>9	0.78 (0.45, 1.36)	0.68 (.037, 1.28)
Religion (Protestant = ref)		
Catholic	1.19 (0.65, 2.17)	1.05 (0.52, 2.14)
Other	1.12 (0.65, 1.93)	0.75 (0.39, 1.43)
GA at baseline (weeks)	0.99 (0.95, 1.04)	
GA at follow-up (weeks)		1.08 (0.81, 1.45)
GA at end of pregnancy (weeks)	0.94 (0.89, 0.98)**	1.12 (1.00, 1.24)**
Self-rated health status (good/very good = ref)		
Neither poor nor good	1.08 (0.65, 1.79)	1.11 (0.64, 1.92)
Very poor/Poor	0.52 (0.19, 1.42)	0.55 (0.18, 1.66)
Has chronic medical condition (no = ref)	0.95 (0.52, 1.75)	1.02 (0.53, 1.95)
Main health decision-maker (herself = ref)		
Husband	0.97 (0.56, 1.67)	0.95 (0.52, 1.74)
Both	1.19 (0.64, 2.22)	1.12 (0.55, 2.28)
Other	0.51 (0.20, 1.28)	0.62 (0.22, 1.74)
Pregnancy complications at baseline (no = ref)	0.66 (0.33, 1.32)	
Pregnancy complications at follow-up (no = ref)		0.69 (0.35, 1.36)
Pregnancy complications developed between baseline & follow-up (no = ref)		
Pregnancy complications developed between baseline & endline (no = ref)	0.77 (0.38, 1.55)	
Pregnancy complications developed between follow-up & endline (no = ref)		0.54 (0.17, 1.68)
Sought care for pregnancy complications by follow-up time (no = ref)		
Sought care for pregnancy complications by endline (no = ref)	1.55 (0.77, 3.11)	1.74 (0.82, 3.71)
Delivery complications (no = ref)	1.10 (0.53, 2.27)	1.31 (0.61, 2.81)
Cesarean (vaginal delivery = ref)	3.09 (0.94, 10.14)*	3.93 (1.15, 13.38)**
Poor/very poor/neither good nor poor self-reported birth experience (good/very good = ref)	1.93 (1.20, 3..11)**	1.46 (0.83, 2.57)
Study site Gem (Asembo = ref)	0.95 (0.59, 1.53)	0.86 (0.51, 1.45)

Notes: OR, odds ratios; CI, confidence interval; all models adjusted for all the factors shown;

*p<0.10;

**p<0.05.

Among the 746 (76.0% of total) women who delivered in a health facility, 75.6% did so in their reported “preferred” place of delivery. In contrast, only 3.1% of women who delivered outside a health facility (88.4% of whom delivered at home; data not shown) intended to do so (Table 5). The most frequently reported reasons for not delivering in the “preferred” place were: not being able to get there in time; health services being too expensive, and not having transportation available when needed. The three most common reasons for women’s choosing their respective health facility as place of delivery were: proximity to home (42.0%), belief that the facility offered the best quality services (17.7%), and considering that specific facility as the most affordable delivery place (17.0%). Among women who delivered outside health facilities, 16.3% report not being able to go elsewhere and 9.5% indicated choosing their respective place of delivery because they knew and selected their birth attendants. The vast majority (93.6%) of women who delivered in a facility but only 32.6% of those who did not, rated their index birth experience as good or very good.

**Table 5:** Reasons for choosing specific place of delivery and self-rated birthing experience.

N = 970	Place of delivery		Intention to deliver in the same place again		
	In a health facility N = 746 (%)	Outside health facility N = 224 (%)	Yes N = 706 (%)	Unsure N = 95 (%)	No N = 169 (%)
Place of delivery was the preferred place (n, %)	564 (75.6)	7 (3.1)	550 (77.9)	13 (13.7)	8 (4.7)
Reasons for not delivering in preferred place of delivery (%)					
Could not get there in time	39	62.2	39.7	48.8	64.6
Health service costs too high	19.8	8.3	25	9.8	4.4
No transportation available	9.3	14.8	5.8	26.8	11.2
Transportation costs too high	6.6	0	7.1	1.2	0
My husband/partner opposed	0.6	0.9	0.6	0	1.2
Other family members opposed	0.6	0	0.6	0	0
No answer	24.2	13.8	21.2	13.4	18.6
Reasons for choosing place of delivery (%)					
Closest to home	42	6.3	39.5	32.6	10.1
Facility offering best service quality	17.7	0.5	18.4	3.2	0
Most affordable place	17	1.8	17.9	4.3	0.6
This is where I went for antenatal care	12.1	0	11.2	6.4	3
Cannot afford going elsewhere	3.8	0.9	4.1	0	0.6
I am treated with respect	1.3	16.3	2.3	11.7	11.4
Did not know where else to go	0.8	4.5	0.4	2.1	6.6
I know the providers/TBA	0.7	9.5	1.4	13.8	1.8
No answer	32.7	63.4	4.8	25.9	66.9
Birth experience rating (%)					
Very poor	1.9	10.3	2	0	13.6
Poor	1.9	29	0.4	3.2	43.2
Neither poor nor good	2.7	28.1	0.9	32.6	27.2
Good	65.6	29	67	60	14.2
Very good	28	3.6	29.8	4.2	1.8

Notes: Chi2-tests p<0.05 for all comparisons between 1) women who delivered in and outside a health facility, and 2) women who would, are unsure, and would not deliver in the same place again.

Overall, 72.8% (n = 706) of all women in the sample would like to deliver their next child in the same place (92.0% of women who delivered in health facilities and 8.9% of those who delivered outside health facilities [data not shown]), 9.8% (n = 95) were unsure about the next delivery place if ever pregnant again, and 17.4% (n = 169) would not want to repeat their index delivery experience (Table 5). Among those who would like to deliver in the same place, 77.9% delivered in their reported “preferred” place as did 13.7% of those who were unsure about delivering in the same place and 4.7% of those who would not deliver in the same place. The most important reason for not delivering in the “preferred” place was not being able to get there on time due to abrupt delivery. When examining the associations between women’s experiences giving birth and their intention to deliver again in the same place, we found that 3.3% of those who would deliver in the same place rated their index birth experience as less than good as did 35.8% of those who are unsure about delivering in the same place again; conversely, 16.0% of those who would definitely not choose the same place for a future delivery rated their index birth experience as good or very good.

## Discussion

This study prospectively examined pregnant women’s intentions and subsequent behaviors vis-à-vis MNH service utilization in the HDSS in Nyanza province, Kenya. We found that an overwhelming majority of pregnant women intend to use antenatal, delivery, postnatal, and neonatal care services in health facilities when asked both early (≤20 weeks gestation) and later (30–35 weeks) in pregnancy. While 98.1% of women did indeed use antenatal care and 88.5% of those with a live birth sought neonatal care for their infants, only 76.9% of women delivered in a health facility and 51.8% obtained postnatal care. All these figures, however, point toward important changes in the use of MNH services in Kenya since 2008 when the last Demographic and Health Survey (DHS) was conducted. At that time, only 44.2% of women in Nyanza province delivered in a health facility and 34.2% used postnatal care [14]. Of note, the present study also documents that women’s knowledge of available free delivery care significantly reduced the odds of discordance between their positive intentions and behaviors regarding delivery in a facility.

Equally important, of the women who delivered in a health facility, 92.0% would deliver in the same facility if pregnant again. At first glance, this shows that the increased demand for facility delivery is being met, and that women are, by and large, satisfied with the quality of services received. Yet, only about three-quarters of women delivered in their “preferred” facility—lack of time or the high costs of services prevented many women from doing so. These same reasons were cited by respondents of the 2008 Kenya DHS who did not deliver in a health facility [14] and were also identified by other studies in similar settings [16–23]. Among women who delivered outside health facilities, 9.5% reported knowing the provider who assisted with their delivery as reason for not delivering in a health facility; women’s familiarity with the provider assisting the birth was noted as a home birth facilitator by other studies [18, 20, 22, 24–28]. Only 32.6% of women who delivered outside health facilities reported good or very good index birth experiences compared to 93.6% of those who delivered in a facility.

Taken together, our findings suggest that a dual approach has the potential to significantly improve MNH in this Kenyan province—women should continue to be encouraged to use MNH services and higher quality services should be offered. It is noteworthy that, the two components of this proposed approach have been shown to be strongly related in Kenya [29]. Women need to be made aware that free maternity services are now available to all Kenyan women in public facilities, while the Ministry of Health places more emphasis on improving the quality of services offered in these facilities. In our study, an overwhelming majority of women in Asembo obtained maternal health services at Lwak Hospital, which is a mission hospital that still charges fees for both maternal and neonatal services; of note, free antenatal care was offered to our study participants in Asembo. Conversely, a majority of women in Gem delivered in public health centres. Notably, fewer women in Asembo than Gem reported having delivery complications, and while pregnancy outcomes did not differ by study site, women’s satisfaction with the services received did—significantly more women in Asembo than in Gem reported having good and very good index birth experiences. Also, women in Gem were significantly more likely than those in Asembo to not follow through on their baseline and follow-up intentions and deliver in a health facility. This may be the result of important differences in the quality of services offered in Asembo versus Gem in terms of infrastructure, provider availability and training, service friendliness, and/or respect shown to patients by the medical personnel. A recent study conducted in the same HDSS found that more women in Asembo than in Gem rated the quality of antenatal services as satisfactory [30]. By and large, users’ dissatisfaction with services was shown to be related to the uncaring, disrespectful, insensitive, and even abusive attitude of health care providers [31, 32], and also with practices that are not culturally compatible [33]. Future studies should examine differences in health service quality between Asembo and Gem while taking into account potential further changes in MNH service utilization.

We identified several predictors of discordance between women’s intentions to use MNH services and their actual use of these services. These predictors can be used to target specific groups of women with messages about the importance and benefits of timely use of MNH services. For example, women at least 35 years of age could be told or reminded about the benefits of delivering in a health facility, given the risks associated with higher maternal age. Single women were more likely than married women not to enact their positive intentions regarding facility delivery and use of postnatal and neonatal care services. When compared to married women in monogamous relationships, those in polygamous relationships had lower odds of not following-up on their intentions to seek postnatal care. Other studies have also shown that husbands/partners play various roles in either facilitating [16, 20, 24] or preventing [34–36] their wives from delivering in facilities. Thus, involving husbands/partners in community-wide or provider-led discussions around MNH service seeking behaviors could improve the use of these services. Yet, in some contexts, a husband’s decision-making power may be exceeded by that of elder women in the same household [24, 34]. Thus, not surprisingly, we also found that having someone other than the woman or her husband as main household health decision-maker decreased the odds of intention-behavior discordance with regard to delivery in a facility. More educated women (were found more likely to carry their facility delivery intentions forward; this is in line with findings from other studies demonstrating that women’s and girls’ education is paramount to improving health care utilization, and, by implication, maternal and neonatal indicators in developing countries [31, 37–40].

The study benefits from having an innovative prospective design, but is not without limitations. Key information collected (i.e. pregnancy/delivery complications, chronic medical conditions, delivery outcomes) was self-reported, thus subject to over or underreporting. However, participants were regularly visited by HDSS community interviewers for demographic (e.g. births, deaths) and health (e.g. infectious diseases, hypertension) surveillance, thus, such misreporting bias was likely low; also, in light of the documented high proportion of women obtaining antenatal and delivery care in this province, such self-reported information is likely based on provider diagnosis or confirmation of the complications and conditions reported. Another limitation of this study was that although most health-related characteristics were similar between the two study sites at baseline, this was not true for certain variables. There were a few baseline differences in women’s health-related characteristics by study site at baseline. The mean gestational age at baseline was lower for women enrolled in Asembo (12.3 weeks) than in Gem (14.0 weeks) because of differences in community mobilization efforts for this study and the manner in which community workers identified pregnant women in the two study sites—household visits represented the only source of identification of pregnant women in Gem, while large pregnancy testing campaigns were also implemented in Asembo villages. Also, due to differences between the two sites whereby the HDSS offers testing and diagnosis in Asembo but not in Gem for a number of infectious diseases, including HIV infection, more women in Asembo than in Gem were aware of their health conditions. This led to a far greater percentage of women in Asembo than in Gem reporting suffering from a chronic medical condition, and to differences in women’s self-rated health status between the two sites. Data on women’s HIV status and presence of other chronic medical conditions were obtained from HDSS records and from women’s reports regarding conditions for which they received care in the past year and current medications taken. However, given the sensitivity of this information, we may have underestimated the proportion of women with chronic medical conditions, including HIV infection. In turn, this may have led to an underestimation of the associations between women having such conditions and their intention-behavior discordance with regard to MNH service utilization. Likely the fact that free antenatal care was offered to all study participants in Asembo but not in Gem may have led to significantly more women in Asembo reporting pregnancy complications at baseline.

These baseline differences could have influenced our identification of predictors of baseline vs follow-up intention-behavior discordance. Notably, we found the same predictors regardless of the timing of intention assessment for one of the outcomes (delivery in a facility), but not for the other two outcomes (use of postnatal and neonatal care). Therefore, using sensitivity analysis, we examined predictors of baseline vs. follow-up intention-behavior discordance with regard to the three outcomes (S1 Table), and found that for all three outcomes, women in Gem were significantly more likely to change their service use intention reports from positive at baseline to negative at the follow-up time (median interval time 18 weeks). However, the three key baseline differences between the two study sites (gestational age, presence of chronic medical conditions, and reports of pregnancy complications) did not significantly predict this change in use intention reports between baseline and follow-up for any of the three outcomes.

In conclusion, this study documents an increasing demand for MNH services in Nyanza province, Kenya, where health indicators are among the worst in the country [14]. The extent to which this higher MNH service demand is adequately met for all socio-economic groups and high quality services are being offered in all types of health facilities (i.e. public, private, mission-based) remains unknown. High quality obstetric care is known to reduce maternal and perinatal morbidity and mortality [41], and a recent meta-review showed that users’ satisfaction with healthcare is key to their continuous use of services [31]. Thus, to avoid seeing a trend reversal in the use of MNH services in Nyanza province in the future, it is paramount that emphasis be placed on the quality of care offered to women seeking these services. An assessment of the quality of MNH services was beyond the scope of our study, but it should be considered by future studies aiming to assess trends in MNH service utilization in Kenya.

## Supporting Information

S1 File This is the Stata file. (DTA)

S1 Table Predictors of discordant baseline-follow-up intention reports regarding facility delivery, postnatal and neonatal care use: Kenya, 2013. Notes: OR, odds ratios; CI, confidence interval; all models adjusted for all the factors shown; *p<0.10; **p<0.05; n/a, predicts the outcome perfectly, thus omitted from the regression model.
(DOCX)

S2 Table This is the Data codebook. (PDF)

## References

[1] WHO/UNICEF/UNFPA/World Bank. Trends in maternal mortality: 1990 to 2013 Estimates by WHO, UNICEF, UNFPA, The World Bank and Bank and the United Nations Population Division. Geneva, World Health Organization, 2014 Available: http://apps.who.int/iris/bitstream/10665/112682/2/9789241507226_eng.pdf?ua=1

[2] Kenya—country statistics. 2012. Available: http://www.unicef.org/infobycountry/kenya_statistics.html

[3] World Bank—Kenya country estimates. 2014. Available: http://data.worldbank.org/indicator/SH.DYN.NMRT?page=2

[4] Loundon I. Death in child-birth: an international study of maternal care and maternal mortality 1800–1950. Oxford University Press, 1992.

[5] de Bernis L, Sherratt DR, AbouZahr C, Van Lerberghe W. Skilled attendants for pregnancy, childbirth and postnatal care. British Medical Bulletin 2003;67: 39–57 14711753 10.1093/bmb/ldg017

[6] Bell J, Hussein J, Jentsch B, Scotland G, Bullough C, Graham W. Improving skilled attendance at delivery: a preliminary report of the SAFE strategy development tool. Birth 2003;30(4): 227–34. 14992153 10.1046/j.1523-536x.2003.00252.x

[7] Thaddeus S, Maine D. Too far to walk: Maternal mortality in context. Social Science & Medicine 1994;38(8):1091–1110. 8042057 10.1016/0277-9536(94)90226-7

[8] Ajzen I. The theory of planned behavior. Organizational Behavior and Human Decision Processes 1991;50:179–211.

[9] Conner M, Armitage CJ. Extending the theory of planned behavior: A review and avenues for further research. Journal of Applied Social Psychology 1998;28:1429–1464.

[10] Conner M, Sparks P. The theory of planned behavior and health behaviors In Conner M. & Norman P. (Eds.); Buckingham: Open University Press Predicting health behavior. 1996:121–162.

[11] Godin G, Kok G. The theory of planned behavior: A review of its applications to health-related behavior. American Journal of Health Promotion 1996;11:87–98. 10163601 10.4278/0890-1171-11.2.87

[12] Randall DM, Wolff JA. The time interval in the intention—behaviour relationship: Meta-analysis. British Journal of Social Psychology 1994;33:405–418.

[13] Sheeran P, Orbell S. Do intentions predict condom use? Meta-analysis and examination of six moderator variables. British Journal of Social Psychology 1998;37:231–250. 9639864 10.1111/j.2044-8309.1998.tb01167.x

[14] Kenya National Bureau of Statistics, Nairobi, Kenya and MEASURE DHS, ICF Macro, Calverton, Maryland, USA. Demographic and Health Survey 2008–09: Final report. 2009. Available: http://www.measuredhs.com/pubs/pub_details.cfm?id=1008&srchTp=home

[15] Andersen RM. Revisiting the behavioral model and access to medical care: does it matter? J Health Soc Behav. 1995; 36(1):1–10. 7738325

[16] Story WT, Burgard SA, Lori JR, Taleb F, Ali NA, Hoque DE. Husbands' involvement in delivery care utilization in rural Bangladesh: A qualitative study. BMC Pregnancy Childbirth. 2012;12(1):28. 22494576 10.1186/1471-2393-12-28 PMC3364886

[17] Doctor HV, Findley SE, Ager A, Cometto G, Afenyadu GY, Adamu F et al Using community-based research to shape the design and delivery of maternal health services in Northern Nigeria. Reprod Health Matters. 2012;20(39):104–112. 10.1016/S0968-8080(12)39615-8 22789087

[18] Parkhurst JO, Rahman SA, Ssengooba F. Overcoming access barriers for facility-based delivery in low-income settings: insights from Bangladesh and Uganda. J Health Popul Nutr. 2006;24(4):438–445. 17591340 PMC3001147

[19] Mwangome FK, Holding PA, Songola KM, Bomu GK. Barriers to hospital delivery in a rural setting in Coast Province, Kenya: community attitude and behaviours. Rural Remote Health. 2012;12:1852 22471588

[20] Sorensen BL, Nielsen BB, Rasch V, Elsass P. User and provider perspectives on emergency obstetric care in a Tanzanian rural setting: A qualitative analysis of the three delays model in a field study. Afr J Reprod Health. 2011;15(2):2. 22590898

[21] Mrisho M, Schellenberg JA, Mushi AK, Obrist B, Mshinda H, Tanner M et al Factors affecting home delivery in rural Tanzania. Trop Med Int Health. 2007;12(7):862–872. 17596254 10.1111/j.1365-3156.2007.01855.x

[22] Seljeskog L, Sundby J, Chimango J. Factors influencing women's choice of place of delivery in rural Malawi—an explorative study. Afr J Reprod Health. 2006;10(3):66–75. 17518132

[23] Pembe AB, Urassa DP, Darj E, Carlstedt A, Olsson P. Qualitative study on maternal referrals in rural Tanzania: decision making and acceptance of referral advice. Afr J Reprod Health. 2008;12(2):120–131. 20695047

[24] Gebrehiwot T, Goicolea I, Edin K, Sebastian MS. Making pragmatic choices: women's experiences of delivery care in Northern Ethiopia. BMC Pregnancy Childbirth. 2012;12(1):113. 23078068 10.1186/1471-2393-12-113 PMC3542090

[25] Izugbara CO, Kabiru CW, Zulu EM. Urban poor Kenyan women and hospital-based delivery. Public Health Rep. 2009;124(4):585–589. 19618796 10.1177/003335490912400416 PMC2693173

[26] Titaley CR, Hunter CL, Dibley MJ, Heywood P. Why do some women still prefer traditional birth attendants and home delivery?: a qualitative study on delivery care services in West Java Province, Indonesia. BMC Pregnancy Childbirth. 2010;10:43 10.1186/1471-2393-10-43 20701762 PMC2928756

[27] Iyengar SD, Iyengar K, Martines JC, Dashora K, Deora KK. Childbirth practices in rural Rajasthan, India: implications for neonatal health and survival. J Perinatol. 2008;28(Suppl 2):S23–S30. 10.1038/jp.2008.174 19057565

[28] Shiferaw S, Spigt M, Godefrooij M, Melkamu Y, Tekie M. Why do women prefer home births in Ethiopia? BMC Pregnancy Childbirth. 2013;13(1):5. 23324550 10.1186/1471-2393-13-5 PMC3562506

[29] Audo MO, Ferguson A, Njoroge PK. Quality of health care and its effects in the utilisation of maternal and child health services in Kenya. East Afr Med J. 2005 11;82(11):547–53. 16463747 10.4314/eamj.v82i11.9407

[30] Ouma PO, van Eijk AM, Hamel MJ, Sikuku ES, Odhiambo FO, Munguti KM et al Reprod Health. 2010;7:1 10.1186/1742-4755-7-1 20429906 PMC2867783

[31] Nair M, Yoshida S, Lambrechts T, Boschi-Pinto C, Bose K, Mason EM et al Facilitators and barriers to quality of care in maternal, newborn and child health: a global situational analysis through metareview. BMJ Open. 2014;4(5):e004749 10.1136/bmjopen-2013-004749 24852300 PMC4039842

[32] Simkhada B, Teijlingen ER, Porter M, Simkhada P. Factors affecting the utilization of antenatal care in developing countries: systematic review of the literature. J Adv Nurs 2008;61:244–60. 10.1111/j.1365-2648.2007.04532.x 18197860

[33] Dogba M, Fournier P. Human resources and the quality of emergency obstetric care in developing countries: a systematic review of the literature. Hum Resour Health 2009;7:7 10.1186/1478-4491-7-7 19200353 PMC2645357

[34] Oyerinde K, Harding Y, Philip A, Garbrah-Aidoo N, Kanu R, Oulare M et al Barriers to Uptake of Emergency Obstetric and Newborn Care Services in Sierra Leone: A Qualitative Study. J Commun Med Health Educ. 2012;2(5):1–8.

[35] Magoma M, Requejo J, Campbell OM, Cousens S, Filippi V. High ANC coverage and low skilled attendance in a rural Tanzanian district: a case for implementing a birth planintervention. BMC Pregnancy Childbirth. 2010;10(13):1–12. 20302625 10.1186/1471-2393-10-13 PMC2850322

[36] Turan JM, Hatcher AH, Medema-Wijnveen J, Onono M, Miller S, Bukusi EA et al The role of HIV-related stigma in utilization of skilled childbirth services in rural Kenya: a prospective mixed-methods study. PLoS Med. 2012;9(8):e1001295 10.1371/journal.pmed.1001295 22927800 PMC3424253

[37] Ahmed S, Creanga AA, Gillespie DG, Tsui AO. Economic status, education and empowerment: implications for maternal health service utilization in developing countries. PLoS One. 2010;5(6):e11190 10.1371/journal.pone.0011190 20585646 PMC2890410

[38] Ono M, Matsuyama A, Karama M, Honda S. Association between social support and place of delivery: a cross-sectional study in Kericho, Western Kenya. BMC Pregnancy Childbirth. 2013;13:214 10.1186/1471-2393-13-214 24261639 PMC4222494

[39] van Eijk AM, Bles HM, Odhiambo F, Ayisi JG, Blokland IE, Rosen DH et al Use of antenatal services and delivery care among women in rural western Kenya: a community based survey. Reprod Health 2006,3**:** 2 16597344 10.1186/1742-4755-3-2 PMC1459114

[40] Hounton S, Chapman G, Menten J, De Brouwere V, Ensor T, Sombié I et al Accessibility and utilisation of delivery care within a Skilled Care Initiative in rural Burkina Faso. Trop Med Int Health 2008, 13(Suppl 1)**:** 44–5. 10.1111/j.1365-3156.2008.02086.x 18578811

[41] Bohren MA, Hunter EC, Munthe-Kaas HM, Souza JP, Vogel JP, Gülmezoglu AM. Facilitators and barriers to facility-based delivery in low- and middle-income countries: a qualitative evidence synthesis. Reprod Health. 2014 9 19;11(1):71 10.1186/1742-4755-11-71 25238684 PMC4247708

