# Supplementary material for: Pregnant Women’s Intentions and Subsequent Behaviors Regarding Maternal and Neonatal Service Utilization: Results from a Cohort Study in Nyanza Province, Kenya
Source: PLoS One. 2016 Sep 13;11(9):e0162017. doi: 10.1371/journal.pone.0162017 (PMC5021282; doi:10.1371/journal.pone.0162017)
Supplement: S1 Table — Notes: OR, odds ratios; CI, confidence interval; all models adjusted for all the factors shown; *p<0.10; **p<0.05; n/a, predicts the outcome perfectly, thus omitted from the regression model. (DOCX) [file pone.0162017.s002.docx]

**S1 Table. Predictors of discordant baseline-follow-up intention reports regarding facility delivery, postnatal and neonatal care use: Kenya, 2013**

| **Characteristics** | **Facility delivery intentions (follow-up vs baseline)**  **N=933** | **Postnatal care intentions (follow-up vs baseline)**  **N=911** | **Neonatal care intentions (follow-up vs baseline)**  **N=952** |
| --- | --- | --- | --- |
|  | OR (95% CI) | | |
| ***Socio-demographic characteristics*** | | | |
| Age-group (25-29=ref)  <20  20-24  30-34  35+ | 1.94 (0.84, 4.50)  0.62 (0.28, 1.37)  2.04 (0.97, 4.31)*   - 1. (0.63, 3.71) | 1.87 (0.86, 4.1)  0.62 (0.30, 1.26)  1.47 (0.72, 3.01)  0.84 (0.34, 2.06) | 2.90 (1.11, 7.56)**  1.00 (0.42, 2.42)  2.39 (0.98, 5.82)*  1.24 (0.39, 3.94) |
| Primipara (multipara=ref) | 1.07 (0.49, 2.37) | 0.81 (0.38, 1.75) | 0.89 (0.37, 2.12) |
| Marital status (married/in union, monogamous=ref)  Married/in union, polygamous  Single | 1.08 (0.52, 2.25)  2.46 (1.28, 4.74)** | 1.18 (0.58, 2.42)  1.93 (1.02, 3.68)** | 0.98 (0.40, 2.39)  2.56 (1.22, 5.36)** |
| Education (5-8 years=ref)  <5  >9 | 0.96 (0.34, 2.69)  0.64 (0.36, 1.16) | 0.71 (0.24,. 2.13)  0.54 (0.30, 0.98)** | 0.52 (0.12, 2.35)  0.70 (0.34, 1.30) |
| Religion (Protestant=ref)  Catholic  Other | 1.47 (0.82, 2.64)  0.88 (0.47, 1.62) | 1.48 (0.84, 2.61)  0.76 (0.42, 1.38) | 1.55 (0.80, 3.02)  0.86 (0.43, 1.71) |
| ***Health-related characteristics*** | | | |
| GA at baseline (weeks) | 0.96 (0.92, 1.00)* | 0.99 (0.94, 1.04) | 0.99 (0.94, 1.04) |
| GA at follow-up (weeks) | 1.41 (1.14, 1.76)** | 1.32 (1.06, 1.65)** | 1.41 (1.11, 1.79)** |
| Self-rated health status (good/very good=ref)  Neither poor nor good  Very poor/Poor | 0.65 (0.36, 1.18)  0.65 (0.23, 1.81) | 0.94 (0.54, 1.63)  0.91 (0.36, 2.31) | 0.83 (0.42, 1.62)  1.43 (0.53, 3.89) |
| Has chronic medical condition (no=ref) | 0.88 (0.42, 1.82) | 0.78 (0.38, 1.60) | 0.46 (0.15, 1.37) |
| Main health decision-maker (herself=ref)  Husband  Both  Other | 0.74 (0.37, 1.46)  1.19 (0.61, 2.33)  0.78 (0.35, 1.73) | 0.86 (0.47, 1.56)  0.62 (0.29, 1.33)  0.80 (0.37, 1.75) | 0.88 (0.41, 1.86)  1.32 (0.62, 2.82)  0.61 (0.24, 1.51) |
| Pregnancy complications at baseline (no=ref) | 1.21 (0.64, 2.26) | 0.77 (0.40, 1.46) | 1.04 (0.51, 2.09) |
| Pregnancy complications developed between baseline & follow-up (no=ref) | 0.31 (0.10, 0.95)** | 0.55 (0.23, 1.32) | n/a |
| Sought care for pregnancy complications by follow-up time (no=ref) | 0.47 (0.13, 1.73) | 0.65 (0.22, 1.92) | n/a |
| Knowledge of free delivery care services at follow-up (no=ref) | 0.94 (0.78, 1.84) |  |  |
| Study site Gem (Asembo=ref) | 2.35 (1.40, 3.95)** | 2.13 (1.30, 3.51)** | 1.78 (0.98, 3.21)* |

Notes: OR, odds ratios; CI, confidence interval; all models adjusted for all the factors shown; *p<0.10; **p<0.05; n/a, predicts the outcome perfectly, thus omitted from the regression model.
