# Supplementary material for: Pregnant Women’s Intentions and Subsequent Behaviors Regarding Maternal and Neonatal Service Utilization: Results from a Cohort Study in Nyanza Province, Kenya
Source: PLoS One. 2016 Sep 13;11(9):e0162017. doi: 10.1371/journal.pone.0162017 (PMC5021282; doi:10.1371/journal.pone.0162017)
Supplement: S2 Table — (PDF) [file pone.0162017.s003.pdf]

Data dictionary – PLOS ONE – MNHCU study

Variable name	Variable label	Variable category value & label
intend_anc_baseline	Baseline antenatal care intention	Yes 1<br>No 0
intend_delfacility_baseline	Baseline facility delivery intention	Yes 1<br>No 0
intend_pnc_baseline	Baseline postnatal care intention	Yes 1<br>No 0
intend_neo_baseline	Baseline neonatal care intention	Yes 1<br>No 0
intend_anc_fu	Follow up antenatal care intention	Yes 1<br>No 0
intend_delfacility_fu	Follow up facility delivery intention	Yes 1<br>No 0
intend_pnc_fu	Follow up postnatal care intention	Yes 1<br>No 0
intend_neo_fu	Follow up neonatal care intention	Yes 1<br>No 0
anc_e	Endline antenatal care use	Yes 1<br>No 0
delfacility_e	Endline facility delivery	Yes 1<br>No 0
pnc_e	Endline postnatal care use	Yes 1<br>No 0
neo_e	Endline neonatal care use	Yes 1<br>No 0
agegr	Age-group (years)	<20 1<br>20-24 2<br>25-29 3<br>30-34 4<br>35+ 5
primimultigravida	Parity	Primipara 0<br>Multipara 1
mstatuspoly	Marital status	Married/in union, monogamous 0<br>Married/in union, polygamous 1<br>Single 2
educgr	Education (years)	<5 1<br>5-8 2<br>>9 3
religion	Religion	Protestant 1<br>Catholic 2<br>Other 3
ga	Baseline gestational age (weeks)	--*continuous*
ga_fu	Follow-up gestational age (weeks)	--*continuous*
ga_e	End of pregnancy gestational age (weeks)	--*continuous*
healthstatus	Self-rated health status	Very poor/Poor<br>Neither poor nor good<br>Good/Very good
chronic	Has (known) chronic medical condition(s)	Yes 1<br>No 0

healthdecisions	Main health decision-maker	Herself 1<br>Husband 2<br>Both 3<br>Other 4
preg_complic_baseline	Self-reported pregnancy complications at baseline	Yes 1<br>No 0
preg_complic_fu	Self-reported pregnancy complications at follow-up	Yes 1<br>No 0
preg_complic_bfu	Self-reported pregnancy complications developed between baseline & follow-up	Yes 1<br>No 0
preg_complic_be	Self-reported pregnancy complications developed between baseline & endline	Yes 1<br>No 0
preg_complic_fe	Self-reported pregnancy complications developed between follow-up & endline	Yes 1<br>No 0
preg_complic	Any self-reported pregnancy complications	Yes 1<br>No 0
care_complic_followup	Sought care for pregnancy complications by follow-up	Yes 1<br>No 0
care_complic_endline	Sought care for pregnancy complications by endline	Yes 1<br>No 0
delcomplic_e	Delivery complications	Yes 1<br>No 0
delmode	Vaginal delivery	Yes 1<br>No 0
pregoutcome_e	Pregnancy outcome	Live birth 4<br>Stillbirth 3
birthexp	Self-reported birth experience	Good/very good<br>2<br>Poor/very poor/neither good nor poor<br>1
kfs_anc_baseline	Knowledge of free antenatal services at baseline	Yes 1<br>No 0
kfs_anc_fu	Knowledge of free antenatal services at follow-up	Yes 1<br>No 0
kfs_del_fu	Knowledge of free delivery services at follow-up	Yes 1<br>No 0
kfs_del_e	Knowledge of free delivery services at endline	Yes 1<br>No 0
ag	Study site	Asembo 0<br>Gem 1
s_delfacility	Non-concordance facility delivery and facility delivery intention at baseline	Yes 1<br>No 0
s_delfacility_fu	Non-concordance facility delivery and facility delivery intention at follow-up	Yes 1<br>No 0
s_pnc	Non-concordance postnatal care and postnatal intention at baseline	Yes 1<br>No 0
s_pnc_fu	Non-concordance postnatal care and postnatal intention at follow-up	Yes 1<br>No 0
s_neo	Non-concordance neonatal care and postnatal intention at baseline	Yes 1<br>No 0

s_neo_fu	Non-concordance neonatal care and postnatal intention at follow-up	Yes 1<br>No 0
repeatbeh_e	Intention to deliver in same place again	Yes 1<br>Unsure 2<br>No 3
prefsdp	Preferred place of delivery	Yes 1<br>No 0
 reason_closestsdp<br>reason_bestqualitysdp<br>reason_mostaffordablesdp<br>reason_ancsdp<br>reason_respectsdp<br>reason_cannotaffordothersdp<br>reason_didnotknowhereelse<br>reason_knowproviderssdp<br>reason_noanswer	*Reason choosing place of delivery*<br>Closest to home<br>Facility offering best service quality<br>Most affordable place<br>This is where I went for antenatal care<br>I am treated with respect<br>Cannot afford going elsewhere<br>Did not know where else to go<br>I know the providers/TBA<br>No answer	 Yes 1<br>No 0
